# Effects of STN-DBS treatment on voice characteristics in patients with Parkinson's Disease

**DOI:** 10.1007/s10072-025-08622-9

**Published:** 2026-01-06

**Authors:** Xinru Hu, Yue Wang, Qi Wang, Jingchao Wu, Haitao Li, Yuanyuan Cheng, Peipei Liu, Yang Yu, Siquan Liang, Jialing Wu

**Affiliations:** 1 Huanhu Hospital Affiliated to Tianjin Medical University, Tianjin Medical University, Tianjin, 300070 China; 2 Department of Rehabilitation, Tianjin Huanhu Hospital, Tianjin, 300350 China; 3 Department of Neurosurgery, Tianjin Huanhu Hospital, Tianjin, 300350 China; 4 Department of Neurology, Tianjin Key Laboratory of Cerebral Vascular and Neurodegenerative Diseases, Tianjin Neurosurgical Institute, Tianjin Huanhu Hospital, Tianjin, 300350 China

**Keywords:** Parkinson's disease, Deep brain stimulation, Subthalamic nucleus, Voice acoustic analysis, Volume of tissue activated

## Abstract

**Objective:**

To evaluate the early changes of voice characteristics after deep brain stimulation (DBS) of subthalamic nucleus (STN) in patients with Parkinson's disease (PD) and to investigate the effect of the volume of tissue activated (VTA) of the STN on voice.

**Methods:**

From April 2024 to October 2024, 23 Parkinson's disease patients who underwent deep brain stimulation of subthalamic nucleus in Tianjin Huanhu Hospital and 22 healthy volunteers were collected. The voice characteristics of 23 Parkinson's disease patients without medication were evaluated preoperative, 3 months and 6 months after surgery. The voice handicap index (VHI) scale was used to evaluate the subjective voice function of patients. LingWAVES voice analysis software was used to collect patient and healthy control data and perform voice acoustic analysis. We further analyzed the correlation between postoperative tissue activation volume and voice characteristics.

**Results:**

The subjective VHI scores improved significantly at 3 months postoperatively (p = 0.025), but there was no significant difference at 6 months postoperatively (p = 0.283). The parameters including dysphonia severity index (DSI), fundamental frequency perturbation (Jitter) and amplitude perturbation (Shimmer) were significantly improved three and six months postoperatively compared with baseline (*p* < 0.05). There was a negative correlation between the improvement rate of DSI and the VTA overlap within limbic STN in the left hemisphere six months postoperatively (R^2^ = 0.4413, *p* < 0.001).

**Conclusion:**

STN-DBS can improve the voice characteristics of Parkinson's disease patients in the early stage after surgery, the improvement rate of DSI was negatively correlated with the VTA overlap within limbic STN in the left hemisphere.

## Introduction

Parkinson's disease is a neurodegenerative disease. Bradykinesia, resting tremor and muscle rigidity are its primary motor symptoms [1]. Apart from core motor symptoms, PD patients also develop dysarthria as the disease progresses. It is estimated that 75%−90% of PD patients suffer from changes in speech and voice [2]. These speech and language changes significantly impact the quality of life for both PD patients and their families [3].

Current treatment approaches for speech disorders include pharmacotherapy, speech therapy, surgery treatment, and vocal fold augmentation [4]. Dopaminergic medications can effectively improve motor function in patients, but their efficacy in treating speech disorders in advanced PD patients is limited [5, 6]. Deep brain stimulation represents the most significant innovation in Parkinson's disease treatment after the discovery of levodopa, effectively improving motor symptoms, quality of life, and non-motor symptoms in PD patients [7, 8]. However, the function of surgery on dysarthria in PD patients is still inconclusive. One study has found that two years after DBS surgery, the procedure did not lead to continuous deterioration in objective assessment of voice functions or patient-reported communication function in PD patients [9]. Some studies have found that PD patients experience deterioration in speech articulation following DBS surgery. Five years after surgery, the overall quality of life improved, but the language problems were poorly managed [10]. Studies have shown that some subtypes of dysarthria are associated with age and disease duration, while other subtypes are related to the spread of electrical currents to corticobulbar fibers [11]. Current research findings on the impacts of DBS on voice in PD patients remain inconsistent internationally [12]. However, current domestic research in this area is limited, and there is insufficient evidence to confirm that STN-DBS leads to dysarthria.

The assessment of dysarthria typically involves both auditory-perceptual and acoustic measures. There are differences in auditory-perceptual and acoustic assessment results in the evaluation of dysarthria in Parkinson's disease [13]. Therefore, this study aims to conduct vocal acoustic assessments in early postoperative period for PD, integrating both subjective and objective analyses to research the impact of surgery on voice characteristics in PD patients and provide evidence for the rehabilitation management of axial symptoms postoperatively.

## Participants and methods

### Participants

A total of 23 PD patients who underwent subthalamic nucleus deep brain stimulation at Tianjin Huanhu Hospital from April 2024 to October 2024 and 22 healthy volunteers undergoing routine physical examinations were enrolled. No statistically significant differences were found in demographic data (age, sex, height, weight) between PD and healthy control (*p* > 0.05) (Table 1).

**Table 1:** Comparison of general characteristics between PD patients and healthy control

Variables	PD patients	Healthy control	*p*
Age, years	62.09 ± 7.11	61.18 ± 7.07	0.671
Males, n (%)	16(69.6)	10(45.5)	0.102
Height, cm	169.35 ± 11.11	167.45 ± 7.37	0.506
Weight, kg	70.09 ± 13.94	66.27 ± 10.74	0.311
Preoperative MDS-UPDRS Ⅲ Med off	43.91 ± 16.24	-	
Preoperative MDS-UPDRS Ⅲ Med on	21.83 ± 11.78	-	
Duration of disease, years	9.48 ± 4.41	-	
H&Y	2.96 ± 0.37	-	
LEDD, mg/d	796.13 ± 380.05	-	

Inclusion criteria for PD patients: (1) Diagnosis of idiopathic Parkinson's disease meeting clinical diagnostic criteria [14]; (2) The thalamic nucleus and deep brain stimulation surgery was performed. All surgeries were performed under local anesthesia. Single-cell recording was used to assist in positioning the sensorimotor area of the nucleus. Once the typical discharge characteristics of the sensorimotor area were observed, the stimulation electrode was implanted. An external stimulator is inserted after the intraoperative test effect is satisfactory (Medtronic 3389 electrode or PINS L301 electrode). (3) Able to cooperate with follow-up assessments and sign informed consent. Exclusion criteria: (1) Severe cognitive impairment, psychiatric disorders, or other conditions preventing completion of examinations; (2) Severe hearing impairment or concurrent diseases causing dysarthria; (3) Patients with surgical contraindications.

### Data collection and assessments

The Voice Handicap Index (VHI) scale was used to assess subjective dysarthria in PD patients. This scale consists of three domains: functional, physical, and emotional, with each domain containing ten items. Each item is rated using the following options: "never," "almost never," "sometimes," and "always," with corresponding scores ranging from 0 to 4. "Never" is scored as 0, "always" as 4, and the remaining options are assigned scores from 1 to 3 [15].

Objective analysis of the patients’ voice characteristics was performed using the LingWAVES voice analysis software. In a quiet room with noise levels below 25 dB, the microphone was positioned 30 cm away from the subject's mouth. The voice assessment was conducted during the "off" period, which was performed 12 h after discontinuing levodopa and other anti-Parkinson's medications [16]. The patient was instructed to take a deep breath and pronounce the vowel/a/for as long as possible at a steady pitch, during which the maximum phonation time (MPT) was recorded. The MPT serves as a reliable indicator of the coordination between the respiratory and the laryngeal systems [9]. The patient is instructed to produce the vowel/a/three times consecutively into the microphone at a comfortable pitch and amplitude, with each production lasting at least 3 s. The machine will automatically calculate the Jitter and Shimmer values. Subsequently, the patient is asked to repeat the vowel/a/three times at the highest possible frequency, sustaining each production for 3 s, from which the machine will automatically derive the lowest intensity (I_low_) and the highest possible frequency (F0_high_). The Dysphonia Severity Index (DSI) is a well-established multiparameter metric for assessing the severity of voice disorders and serves as a reliable method for quantifying dysphonia. The DSI is computed using a specific formula. DSI = 0.13MPT + 0.0053F0_high_−0.26I_low_−1.18jitter + 12.4 [17]. The preoperative baseline, three months postoperatively, and six months postoperatively were evaluated when the patient was not taking levodopa medication. The improvement rate of DSI = (preoperative DSI-postoperative DSI)/preoperative DSI.

Motor ability was assessed by the Movement Disorder Society Unified Parkinson's Disease Rating Scale Part Ⅲ (MDS-UPDRS Ⅲ), with a total score of 108 points. Higher scores indicate more severe motor symptoms of Parkinson's disease. The motor function assessment was conducted during the "off" period, which was performed 12 h after discontinuing levodopa and other anti-Parkinson's medications. The improvement rate of MDS-UPDRS III = (preoperative MDS-UPDRS Ⅲ-postoperative MDS-UPDRS Ⅲ)/preoperative MDS-UPDRS Ⅲ. To acknowledge the cognitive condition, the Mini-Mental State Examination (MMSE) was used, which include orientation, attention, memory, and other aspects. The total score is 30 points, and the lower the score, the worse the cognition. The scoring criteria were adjusted according to the corresponding level of education.

### Electrode localization and volume of tissue activated

Preoperative MRI and postoperative CT were linear co-registration and nonlinear normalization using advanced normalization tools to the Montreal Neurological Institute (MNI) NLin2009bAsym template space [18]. To graphically display electrode positions, 2D slices were rendered using a 7-T 100-μm ex vivo human brain MRI template as background images. The reconstruction process is carried out in the Lead-DBS Toolbox (version 3.0) [19]. The volume of tissue activated (VTA) of the STN is calculated from the stimulation parameters [20]. The Lead-Group was used to display 3D images of patients in the standard space [21]. All calculations were performed by MATLAB 2023a.

### Statistical methods

Statistical analysis of the data was performed using SPSS 27.0 statistical software. Count data used the chi-square test, expressed as case numbers. The measurement data were tested for normal distribution and homogeneity of variance, expressed as mean ± standard deviation (x̅ ± s). For inter-group comparisons, independent sample *t*-tests were used, while intra-group comparisons used repeated measures ANOVA. Data not conforming to a normal distribution were expressed as interquartile range (IQR), with inter-group comparisons using the Mann–Whitney U test and intra-group comparisons using the Wilcoxon signed-rank test. After surgery, the Pearson correlation test was used to analyze the correlation between the variables. A *p*-value of < 0.05 was considered statistically significant.

## Results

### Voice assessment results

Preoperative DSI, Jitter, and Shimmer indices showed statistically significant differences compared to the healthy control (*p* < 0.05). However, no statistically significant differences were found between the postoperative values and the healthy control (*p* > 0.05). There were statistically significant differences between patients before and after MPT surgery and healthy controls (*p* < 0.05). No statistically significant differences were found in the lowest intensity and the highest possible frequency between patients (pre- and post-operation) and the healthy control (*p* > 0.05) (Table 2).

**Table 2:** Clinical characteristics of healthy control and Parkinson's disease patients at baseline, 3 months and 6 months postoperatively

Variables	Healthy Control (HC)	Preoperative baseline	3 months postoperatively	6 months postoperatively	HC vs Preoperative baseline		HC vs 3 months postoperatively		HC vs 6 months postoperatively	
					Mean Difference (95%CI)	*p*	Mean Difference (95%CI)	*p*	Mean Difference (95%CI)	*p*
DSI	−2.10 ± 1.68	−3.57 ± 2.02^a^	−2.29 ± 2.18	−2.24 ± 2.33	1.38 (0.16,2.61)	0.027	0.10 (−1.12,1.33)	0.865	0.05 (−1.18,1.27)	0.938
Jitter	0.12(0.10,0.18)	0.20(0.14,0.67)^a^	0.11(0.09,0.21)	0.12(0.08,0.19)	−0.08 (−0.31,−0.02)	0.008	0.01 (−0.04,0.05)	0.750	0.01 (−0.04,0.05)	0.649
Shimmer	10.93 ± 4.08	13.31 ± 4.54^a^	9.95 ± 2.34	10.94 ± 3.91	−2.31 (−4.56,−0.06)	0.044	1.05 (−1.20,3.30)	0.357	0.06 (−2.19,2.31)	0.956
MPT	16.50 ± 6.79	12.02 ± 7.20^a^	12.21 ± 4.84^a^	12.80 ± 4.62^a^	4.41 (0.88,7.95)	0.015	4.23 (0.69,7.76)	0.020	3.63 (0.10,7.17)	0.044
the lowest intensity	67.89 ± 5.82	68.83 ± 7.72	66.02 ± 7.13	65.73 ± 8.09	−0.85 (−5.15,3.44)	0.694	1.96 (−2.34,6.25)	0.368	2.24 (−2.06,6.54)	0.303
the highest possible frequency	222.37 ± 90.14	197.81 ± 55.52	202.69 ± 58.29	226.71 ± 56.10	13.87(−25.05,52.79)	0.481	8.99(−29.94,47.91)	0.648	−15.03(−53.95,23.89)	0.445

compared to healthy control, ^a^*p* < 0.05.

**Fig. 1:**
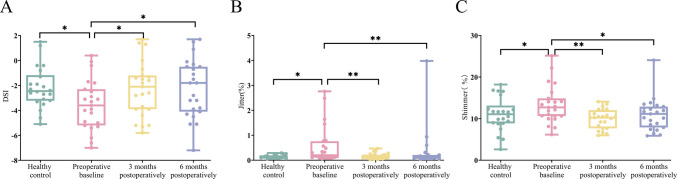
A-C. Comparison of voice assessment results between the healthy control group and Parkinson's disease patients at preoperative, 3-month and 6-month postoperatively (*p < 0.05, **p < 0.01)

The subjective Voice Handicap Index scores showed statistically significant improvement at 3 months postoperatively compared to baseline (*p* = 0.025), but no significant difference was observed at 6 months postoperatively (*p* = 0.283). For objective measures, the Dysphonia Severity Index demonstrated statistically significant improvement at both 3 months (*p* = 0.039) and 6 months (*p* = 0.031) postoperatively compared to preoperative baseline. Similarly, Jitter exhibited statistically significant improvement at 3 months (*p* = 0.009) and 6 months (*p* = 0.016) postoperatively. Shimmer showed statistically significant improvement at 3 months (*p* = 0.004) and 6 months (*p* = 0.037) postoperatively. No statistically significant differences were observed in maximum phonation time, the lowest intensity, and the highest possible frequency at either 3 or 6 months postoperatively compared to preoperative baseline (*p* > 0.05) (Table 3) (Fig. 1).

**Table 3:** Clinical characteristics of Parkinson's disease patients at baseline, 3 months and 6 months postoperatively

Variables	Preoperative baseline	3 months postoperatively	6 months postoperatively	Preoperative baseline vs 3 months postoperatively		Preoperative baseline vs 6 months postoperatively	
				Mean Difference (95%CI)	*p* value	Mean Difference (95%CI)	*p* value
MDS-UPDRS Ⅲ	43.91 ± 16.24	22.61 ± 7.45^b^	18.13 ± 8.51^b^	21.30(14.58,28.03)	< 0.001	25.78(19.06,32.51)	< 0.001
MMSE	25.83 ± 2.15	26.48 ± 2.11	26.43 ± 2.06	−0.65(−1.89, 0.59)	0.297	−0.61(−1.85, 0.63)	0.331
VHI	29.87 ± 14.36	21.04 ± 11.29^b^	25.04 ± 15.76	8.83(0.63,17.02)	0.025	4.83(−3.37,13.02)	0.283
DSI	−3.57 ± 2.02	−2.29 ± 2.18^b^	−2.24 ± 2.33^b^	−1.28(−2.49,−0.07)	0.039	−1.33(−2.55,−0.12)	0.031
Jitter	0.20 (0.14,0.67)	0.11 (0.09,0.21)^b^	0.12 (0.08,0.19)^b^	0.09(0.03,0.29)	0.009	0.08(0.02,0.26)	0.016
Shimmer	13.31 ± 4.54	9.95 ± 2.34^b^	10.94 ± 3.91^b^	3.36(1.13,5.58)	0.004	2.37(0.15,4.60)	0.037
MPT	12.02 ± 7.20	12.21 ± 4.84	12.80 ± 4.62	−0.18(−3.68,3.31)	0.917	−0.78(−4.28,2.72)	0.659
the lowest intensity	68.83 ± 7.72	66.02 ± 7.13	65.73 ± 8.09	2.81(−1.44,7.06)	0.192	3.09(−1.16,7.34)	0.151
the highest possible frequency	197.81 ± 55.52	202.69 ± 58.29	226.71 ± 56.10	−4.89 (−43.37,33.60)	0.801	−28.90 (−67.39,9.59)	0.139

compared to baseline, ^b^*p* < 0.05.

### Electrode localization and volume of tissue activated model

**Fig. 2:**
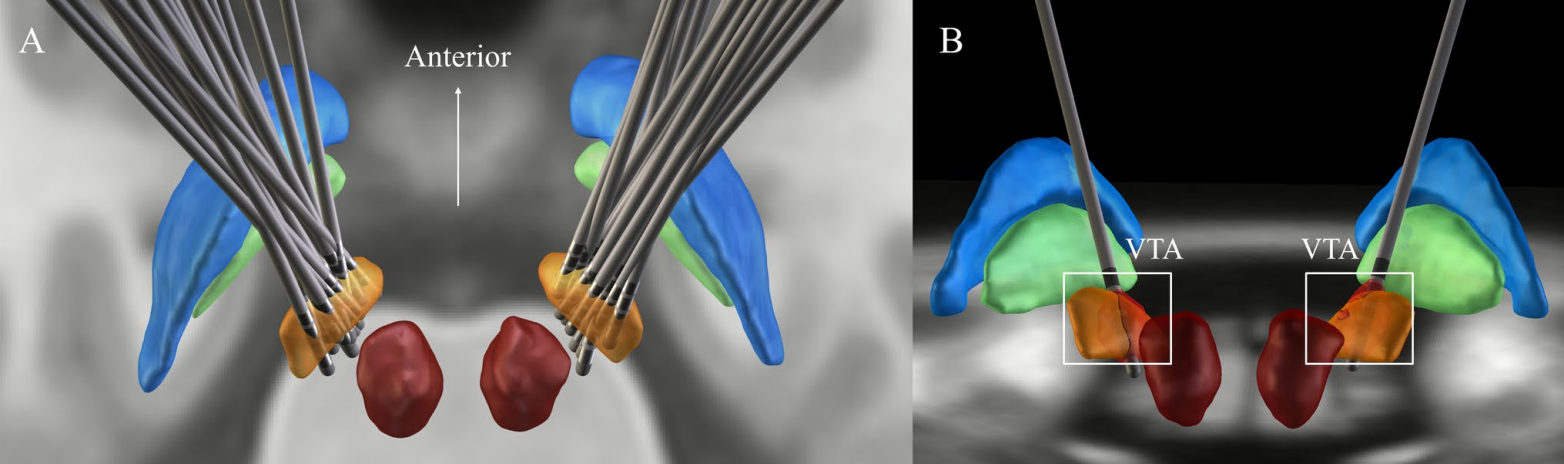
**A**. 3D reconstruction of all electrodes in common atlas space. **B**. The volume of tissue activation (VTA) is the red area outlined

**Fig. 3:**
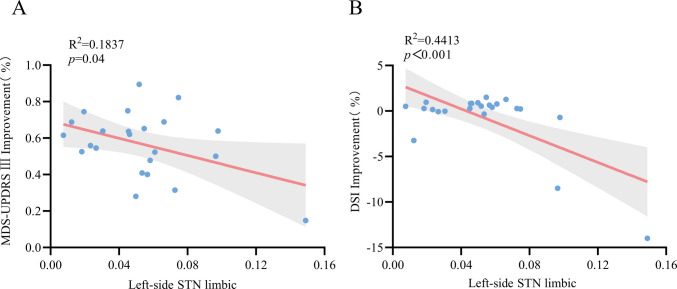
**A**. Correlation between the VTA overlap within left-side limbic of STN and UPDRSⅢ improvement. **B**. Correlation between the VTA overlap within left-side limbic of STN and DSI improvement

Preoperative MRI and postoperative CT scans were reconstructed for 23 patients (Fig. 2A). The volume of tissue activated (VTA) was calculated based on stimulation parameters (Fig. 2B). Results of VTA within motor, associative and limbic parts of the STN six months postoperatively were calculated from the stimulation parameters. The Pearson correlation test was used to analyze the relationship between the improvement of MDS-UPDRSⅢ, improvement of DSI and VTA within motor, associative and limbic parts of the STN six months postoperatively. The results show that the improvement rate of MDS-UPDRS III at six months postoperatively was negatively correlated with and the VTA overlap within limbic STN in the left hemisphere six months postoperatively (R^2^ = 0.1837, *p* = 0.04). There was a negative correlation between the improvement rate of DSI and the VTA overlap within limbic STN in the left hemisphere six months postoperatively (R^2^ = 0.4413, *p* < 0.001). (Fig. 3).

## Discussion

Parkinson's disease is a prevalent neurodegenerative disease. While levodopa medications can initially alleviate symptoms, disease progression often leads to drug-related complications and shortened efficacy duration. Consequently, at advanced stages, deep brain stimulation offers better benefits to patients [8]. The pyramidal tract activation caused by the spread of electrical stimulation elicits side effects associated with speech disorders [22, 23]. The deterioration of voice quality following deep brain stimulation may be caused by disease advancement, the stimulation process, surgical trauma, or a blend of these elements. Discrepancies in assessment techniques, protocols, and stimulation parameters have led to a lack of consensus in current linguistic findings. To understand that the patients’ dysarthria is owing to worsening symptoms or stimulating side effects, the patients need to be evaluated continuously.

In this study, we advocate for early voice assessment following STN-DBS in PD. By examining the changes in voice characteristics of 23 Parkinson's disease patients preoperatively, at 3 months and 6 months postoperatively, we are aimed at clarify the function of STN-DBS on voice in the early stage. This analysis will serve as a foundation for enhancing the holistic care and rehabilitation strategies for dysarthria in patients.

VHI is an assessment that can be used after DBS to evaluate the impact of patient speech on quality of life. The study revealed an improvement in subjective voice function at 3 months postoperatively compared with baseline. However, this improvement was not statistically significant at 6 months postoperatively. In contrast, motor function, evaluated through UPDRS Part III, continued to enhance at both 3 and 6 months postoperatively. The findings indicated that while subjective voice function showed enhancement at 3 months post-surgery, it tended to revert to baseline levels over time, with a less pronounced improvement compared to motor function. Onder H et al. investigated patients undergoing STN-DBS and a pharmacologic control group. Their assessment using the VHI scale suggested no substantial evidence supporting the notion that DBS exacerbates speech disorders in patients [24]. Wertheimer J et al. conducted a comparative analysis between surgical and nonsurgical patients, revealing more pronounced subjective voice disorders in the former group. The study indicated that dysarthria issues in individuals undergoing deep brain stimulation surgery were predominantly associated with the surgical procedure rather than age or disease duration. However, the study's reliance on self-reported assessments by patients, without accounting for potential cognitive factors, introduces certain limitations [25]. The VHI scale not only evaluates the voice function of patients, but also involves emotion, which is easily affected by subjective emotions. Therefore, the subjective scale evaluation has certain limitations, and objective indicators are still needed for targeted evaluation.

The study incorporated voice parameters such as DSI, Jitter, and Shimmer. These parameters exhibited enhancement at 3 and 6 months postoperatively. Preoperative parameters demonstrated statistical significance in comparison to a healthy control group. However, postoperatively, there was no statistical significance noted when compared to the healthy control group. This suggests ongoing improvement in these voice parameters among patients within six months following STN-DBS, with the voice indices showing a tendency towards approaching those of a healthy control group. While STN-DBS can ameliorate patients' motor function, research outcomes regarding its impact on speech function are inconsistent. Mate MA et al. previously used microphone recordings to find improvement in Jitter index from preoperative baseline in patients one month after surgery [26]. Tanaka Y et al. found that DBS stimulation aggravated Jitter and Shimmer indicators more than half a year after surgery [27]. In this study, the voice parameters including DSI, Jitter and Shimmer continued to improve six months after operation, and the results of voice parameters were inconsistent six months after operation or because the patient's disease duration was shorter than that of Tanaka Y et al.

Correlation test was used to analyze the correlation between motor improvement rate, voice improvement rate and motor, association and limbic overlap of STN respectively six months postoperatively. The improvement rate of MDS-UPDRSIII and the improvement rate of DSI at six months after surgery were correlated with the overlap between the VTA overlap within left-side limbic STN six months postoperatively, respectively. The increase of VTA within the limbic part of the left STN was associated with the aggravation of dysarthria. Similarly, one study using unilateral electrode stimulation has found that the left STN is associated with decrease of language fluency. The neural mechanisms responsible for alterations in speech fluency following deep brain stimulation surgery remain ambiguous, potentially implicating disturbances within the basal ganglion-thalamic cortical networks. Neuroimaging researches consistently highlight the involvement of the left inferior gyrus in tasks related to verbal fluency [28]. We observed that certain patients exhibited minimal enhancement in motor symptoms relative to their preoperative baseline. These individuals also had a lengthier preoperative disease course, more pronounced motor symptoms, and required higher stimulation parameters, resulting in heightened dysarthria. Numerous studies have demonstrated that deep brain stimulation can induce alterations in speech and voice. Nevertheless, the precise mechanism remains ambiguous. Several theoretical hypotheses have been proposed to elucidate the phenomenon of voice modifications subsequent to subthalamic nucleus stimulation. These include the spread of stimulation to neighboring structures, somatotopic organization within the STN based on function, or alterations in skeletal motor function in alignment with the general motor response. One conjecture suggests that the propagation of electrical fields to neighboring structures, such as cortical regions or fibers of the internal capsule, may disrupt speech patterns by influencing the musculature of the larynx and lips [29].

Dopamine replacement therapy is essential in managing Parkinson's disease. With the progression of the disease, the development of motor fluctuations and dyskinetic movements should be managed by surgical, rehabilitation, and other specialized teams [30]. After acute levodopa impulse test, motor function improved significantly in PD patients, but speech and voice did not [5, 6, 31]. Bobin M et al. study reckoned that STN-DBS can carefully arrange the stimulation program according to the central stimulation characteristics, and significantly improve the voice quality [32]. This study revealed enhancements in certain voice parameters in early postoperative period, with STN-DBS stimulating at lower stimulation parameters. However, with the enhancement of stimulation voltages postoperatively, it will lead to pyramidal tract stimulation-induced side effects, inducing dysarthria. Hence, we are going to keep assessing to examine the impact of surgery on subjective and objective voice parameters in the patients. Owing to the persistent motor impairment, patients have suffered respiratory muscle weakness, abdominal muscle rigidity, and diminished vital capacity. Physical treatments like Lee Silverman Voice Treatment and respiratory muscle strengthening exercises have shown promise in alleviating dysarthria. These interventions have the potential to enhance maximum phonation time and sound pressure levels after training [33, 34]. It is recommended that the suboptimal indices identified in this study should be addressed through subsequent speech and respiratory rehabilitation interventions aimed at enhancing all aspects of patients' speech function comprehensively.

There are several limitations in this study. Primarily owing to its small sample size, which diminishes its statistical power and the sample size will be further expanded in the future. Furthermore, the study is constrained by its observational design, which introduces limitations. The absence of randomization increases the risk of confounding bias, such as variations in the initial health conditions of patients receiving surgical interventions. Being an observational study, there is also a potential for selection bias, exemplified by the predominantly local sample composition, thereby restricting the generalizability of the findings to a broader demographic.

Tripoliti E et al. observed a decline in speech intelligibility happening one year postoperatively [35]. The conclusions of studies on voice changes after deep brain stimulation are inconsistent (Table 4). Our study will keep assessing the vocal characteristics in our cohort one year postoperatively and investigate the onset of dysarthria. Excessive communication between the cortex and thalamus in PD patients is believed to be engaged significantly in the development of aberrant subthalamic beta activity. Both dopamine and STN-DBS exhibit similar effects on the communication pathways between the cortex and basal ganglia, leading to a decrease in hyperconnectivity in high beta frequencies, which are mediated by the hyperdirect pathways [36]. DBS-induced increased connectivity of cortico-striatal, thalamo-cortical and direct pathways and decreased connectivity of ultra-direct pathways [37]. At present, there is no unified conclusion on the mechanism of DBS stimulation on circuit which is associated with dysarthria, suggesting that further research should be conducted in order to better understand the correlation between DBS stimulation and speech disorders, and to explore the underlying mechanisms.

**Table 4:** Summary table of previous studies

Author	Sample size	Medium Follow up time	Voice parameters	Results
Tripoliti E et al. [35]	32	1 year	Speech intelligibility (% of words understood)	Most patients exhibiting decline of speech intelligibility
Mate MA et al. [26]	25	1 month	fundamental frequency (F0), Jitter, Shimmer	Nearly all acoustic analysis parameters improved after DBS, and the factor Jitter was most sensitive
Wertheimer J et al. [25]	758	5 years	The voice handicap index (VHI)	With the DBS group reporting more severe voice disorder
Tanaka Y et al. [27]	68	≥ 6 months	the GRBAS scale The voice handicap index (VHI), Jitter, Shimmer	VHI, Jitter, Shimmer in the DBS group were significantly worse than the medical therapy group in female patients
Jorge A et al. [29]	14	7 months	visual analog scale (VAS), sound pressure level (SPL), F0, etc	There were no significant differences between pre- and postoperative measures in voice outcomes
Onder H et al. [24]	66	4 years	The voice handicap index (VHI)	Did not find convincing evidence supporting the increased risk of speech disturbance with STN‑DBS therapy

## Conclusion

Ultimately, this study illustrates that subthalamic nucleus deep brain stimulation enhances the voice handicap index scores three months postoperatively and some voice parameters of patients showed sustained improvement at three and six months postoperatively. Furthermore, the improvement rate of DSI was negatively correlated with the VTA overlap within limbic STN in the left hemisphere six months postoperatively. These findings support the ongoing alleviation of voice outcomes in the initial postoperative period, thereby establishing a basis for subsequent assessments and rehabilitation interventions.

## Data Availability

After a reasonable request, the datasets generated during the current study are available from the corresponding author.
